# Peripheral Arterial Disease in Human Immunodeficiency Virus Infection

**DOI:** 10.1007/s11883-026-01410-6

**Published:** 2026-04-24

**Authors:** Elizabeth Sarah Mayne, Susan Louw, Michael Freeman, Nicholas Funderburg, Ismail Cassimjee, Anthony Leland Hamilton Mayne

**Affiliations:** 1 Division of Immunology, Department of Pathology, Faculty of Health Sciences, University of Cape Town, Office 3B22, Falmouth Building, Anzio Road, Observatory, Cape Town, 7708 South Africa; 2 National Health Laboratory Service, 1 Modderfontein Road, Johannesburg, South Africa; 3 Department of Molecular Medicine and Haematology, School of Pathology, Faculty of Health Sciences, University of Witwatersrand, 7 York Road, Parktown, Johannesburg, 2193 South Africa; 4 Department of Medicine, Division of Infectious Diseases and HIV Medicine, Case Western Reserve University, Case Western Reserve School of Medicine, Cleveland, OH 44106 USA; 5 Division of Medical Laboratory Science, School of Health and Rehabilitation Sciences, The Ohio State University, Columbus, OH 43210 USA; 6 Department of Surgery, Division of Vascular Surgery, Charlotte-Maxeke Johannesburg Academic Hospital, University of Witwatersrand, 7 York Road, Parktown, Johannesburg, 2193 South Africa; 7 Department of Philosophy, University of Johannesburg, Auckland Park Campus, Johannesburg, 2006 South Africa

**Keywords:** Peripheral arterial disease, Human immunodeficiency virus, Chronic inflammation, Atherosclerosis

## Abstract

**Purpose of review:**

Human immunodeficiency virus infection (HIV) is a chronic inflammatory and pro-atherosclerotic condition although the contribution of HIV to the development of peripheral arterial disease (PAD) is unclear. PAD is more prevalent and extensive in sub-Saharan African individuals.

**Recent Findings:**

A total of 38 studies reporting results from a median of 298 people living with HIV (PLWH) were included. The majority of studies (24) reported an association between HIV infection and PAD. Study design was heterogeneous. Traditional cardiovascular (tobacco smoking, diabetes mellitus, dyslipidaemia and hypertension) and HIV-associated (CD4 + T cell count, advanced HIV infection, viral load and antiretroviral therapy) risk factors were inconsistently reported. The most commonly reported risk factors were advanced age (odds ratio 1.09–4.66), HIV infection (odds ratio 1.22–4.92) and tobacco smoking (odds ratio range 1.08–5.96). Only 9 studies were conducted in sub-Saharan Africa.

**Summary:**

HIV infection is a risk factor for PAD although heterogeneous study design makes comparison of risk factors difficult. Further studies are required to understand the pathogenesis of PAD in PLWH, to identify biomarkers which may have diagnostic or prognostic significance in this group of patients.

## Introduction

Peripheral arterial disease (PAD) is an atherosclerotic, vascular condition which can be asymptomatic or present with pain in a muscle group on exertion (claudication). In its severest form, it may present with chronic limb-threatening ischaemia (CLTI). This is characterised by severe limb pain at rest, non-healing ulceration or necrotic tissue loss (gangrene) [1, 2]. Despite advances in revascularisation as a therapy for CLTI, approximately 25% of patients in specialised care will require amputation [3, 4]. Mortality is high in patients with CLTI (25% at 1 year) [4], and may reach over 35% in patients requiring amputation [3]. An ankle-brachial index (ABI) below 0.9 is diagnostic for PAD, and patients with CLTI have ABIs less than 0.4. An ABI value above 1.4 suggests a non-compressible artery which may be associated with arterial calcification [5]. Results of large epidemiological studies suggest that approximately 8.5 million patients are diagnosed with PAD in the United States with a cost of hospitalisation exceeding $6 billion per annum [3]. In sub-Saharan Africa, the prevalence of PAD varies but in populations with risk factors like diabetes mellitus, as many as 52% of patients have PAD [6]. PAD is more common and typically more severe in patients of Black African descent [7].

Traditional risk factors for PAD and other atherosclerotic cardiovascular diseases include hyperlipidaemia, tobacco smoking, diabetes mellitus, and hypertension as well as a family history of atherosclerotic disease [1, 2]. PAD includes lesions in any component of the arterial tree distal to the aorta although upper limb atherosclerotic disease is much less common than lower limb disease.

Initial treatment of PAD focuses on treating the underlying risk factors with aggressive management of blood glucose, hypertension and dyslipidaemia, antiplatelet therapy, and encouraging smoking cessation [1]. Revascularisation is reserved for patients with CLTI, and may involve endovascular techniques such as angioplasty and stenting or open techniques such as bypass with a vein conduit [8]. Finally, should revascularisation fail and in the context of tissue loss, it may be necessary to amputate the limb distal to the critical obstruction. Meta-analyses suggest that there is improved vessel patency at 1 year with open revascularisation with higher degrees of technical success although morbidity and mortality risk at 1 month and major amputation risk at 1 year are also increased [9]. The incidence of amputation is typically higher in low- and middle income countries (LMIC) [6, 10] with possible reasons including poor management of risk factors like hypertension and delayed presentation.

Human immunodeficiency virus (HIV) is a lentivirus which infects cells which express the CD4 receptor, predominantly CD4 + T cells and macrophages. HIV infection is a pro-thrombotic and pro-atherosclerotic state secondary to chronic inflammation, endothelial activation and dysfunction and metabolic dysregulation linked both to the disease, itself, and to antiretroviral therapy (ART) [11]. The Strategic Management of Antiretroviral Therapy (SMART) study indicated that patients in whom ART was interrupted were at increased risk of death but specifically from cardiovascular disease and this risk was associated with elevated levels of both interleukin-6 and D-dimers (fibrin degradation products) [12]. With improved access to ART, the risk of opportunistic infections in people living with HIV (PLWH) has reduced although HIV remains a risk factor for HIV-associated non-communicable comorbidities (NCC) particularly in aging PLWH [13]. HIV infection is associated with an increased risk of venous thromboembolic disease [14, 15], microvascular disease (including disseminated intravascular coagulation and thrombotic thrombocytopenic purpura) [16–19] and arterial thrombosis including myocardial infarction, and cerebrovascular accidents although the contribution of HIV to the development of PAD is less well-defined [13].

HIV infection impacts the endothelial cells which are the primary regulators of both inflammation and coagulation [11]. PLWH have increased levels of markers of endothelial dysfunction and/or activation like intercellular and vascular cell adhesion molecule-1 (ICAM-1 and VCAM-1), Platelet and Endothelial selectins (P- and E-selectin) and soluble thrombomodulin, and markers of coagulation cascade dysregulation like von Willebrand factor (VWF) [14]. PLWH have increased levels of inflammatory markers including interleukin (IL)−1 and 6 and tumour necrosis factor-alpha and lipid mediators of inflammation [14]. These factors are all associated with recruitment of pro-inflammatory macrophages and platelet activation [20, 21] and with the development of atherosclerotic plaques in the arterial system.

The diagnosis, optimal management strategies and prognosis of PAD in PLWH is unclear although surgical outcomes are reported as inferior in PLWH and are impacted by virological factors including persistent viraemia, stage IV clinical disease or acquired immunodeficiency syndrome (AIDS) and the extent of immunosuppression [10, 22, 23].

In order to characterise PAD in PLWH, a scoping review was undertaken.

## Methods

**Fig. 1:**
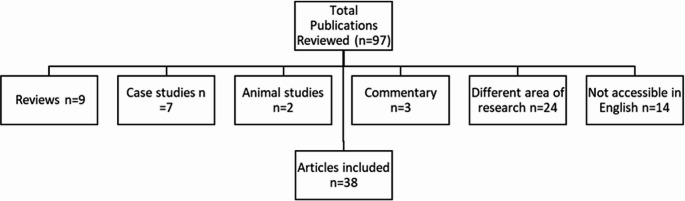
PRISMA scheme for article selection

A search was conducted on PUBMED, MEDLINE, SCOPUS and Google Scholar using the Medical Subject Heading (MeSH) terms: “Human immunodeficiency virus” and “peripheral arterial disease” or “lower extremity arterial disease” between December 2002 and December 2024. Ninety-seven articles were identified on these databases published between December 2002 and June 2023. Grey literature, single case studies, commentaries, reviews, studies performed in animals, studies not available in English language and primarily in vitro studies were excluded (Fig. 1). A final total of 38 publications was included.

Where available adjusted odds ratios or hazard ratios were reported if significant (*p* < 0.05) and relative risks were converted to odds ratios using an assumed baseline prevalence of 10%. Owing to the heterogeneity of sample populations, odds/hazard ratios were not weighted for number of participants.

## Results

The summary of the findings of the included papers is presented in Table 1. Of the 38 articles reviewed, 35 were descriptive, observational studies, 2 measured biomarkers in PLWH and PAD [49, 56] and 1 investigated the influence of exercise on the diagnosis of PAD [28]. In 24 of the 38 articles, the prevalence of PAD was found to be increased in PLWH. Significant heterogeneity was, however, noted in the study population, the inclusion of PWoH (people without HIV - control groups) and the diagnostic criteria applied to make the diagnosis of PAD. A median of 298 PLWH were included in the studies reviewed (range 42–82 426) with 14 studies including only ART-treated PLWH, 16 including ART-naïve and ART-treated PLWH and 2 including only ART-naïve PLWH. In 2 studies, the impact of specific antiretroviral agents, tenofovir, abacavir and efavirenz, was studied in PLWH [34, 58]. In 6 studies, the treatment status of the participants was not discussed. A control cohort was included in 21 of 38 studies (55%) and 11 of the studies were prospective, longitudinal cohort studies. In 13 out of 38 studies (34%), ABI was not measured and in those where ABI was measured, only 6 studies provided values for the complete range of measurement (defined as low, borderline, normal and high). The use of a “borderline” ABI of between 0.9 and 0.99 was inconsistently applied although in a study of the impact of exercise on PAD diagnosis, it was noted that post-exercise ABIs reduced to below 0.9 in 4 participants [33]. Overall, the reported prevalence of PAD in PLWH ranged from 0,09–24,6% with a median of 4,68%.

**Table 1:** Summary of study findings of peripheral arterial disease in people living with HIV

Author (year)	Study design (location)	Cohort description							Traditional cardiovascular risk factors in HIV-infected participants					Main findings
		HIV-infected cohort						HIV-uninfected control group	Diabetes Mellitus *n*(%)	Tobacco smoking *n* (%)	HT *n* (%)	Obesity *n*(%)	Hyper-lipidaemia *n*(%)	
		Total number	Mean Age (years)	Sex men, *n*(%)	CD4 + T cell count (cells/mm^3^)	Aviraemic *n*(%)/Viral load cps/ml	On ART *n* (%)							
2002 Van Marle J et al.[24]	Clinical Audit (South Africa)	42	37	38 (90.5)	CD4 T+ cell count: <200 cells/ul (*n* = 11), 200–500 cells/ul (*n* = 24) and > 500cells/ul (*n* = 7)	NS	3 (7)	0	*n* = 0	21 (50)	NS	NS	0	Outcome of surgical intervention for PAD was influenced by HIV stage (high viral load or low CD4 + T cell count), hypoalbuminaemia and the presence of opportunistic infections
2007 Sharma A et al.[25]	Multicenter observational (United States)	238	39.6	0 (0)	437	83 (34.9)	164 (68.9)	161	23 (9.7%)	104 (43.5%)	55 (23.4%)	78 (32.8%)	14 (5.9%)	Only 2 patients with ABI < 0.9. ABI > 1.4 associated with tobacco use (OR 2.53) and decreased body-mass index (OR 11).
2007 Botes K et al[22]	Clinical Audit (South Africa)	109	40	88 (80.7)	Aneurysmal disease *n* = 24 (22% - mean CD4 + T cell count 323.2 cells/ul) Occlusive disease *n* = 66 (61% - mean CD4 + T cell count 300.2 cells/ul)	NS	0	0	*n* = 2 (2%)	NS	*n* = 23 (21%)	NS	*n* = 3 (2.2%)	Peri-operative mortality for aneurysmal disease was 10.6% versus 3.6% for occlusive disease (*p* = 0.264). Long-term mortality was significantly worse (*p* = 0.049) for patients with aneurysmal disease. Failure of revascularization in > 75%
2008 Bernal E et al.[26]	Descriptive cross-sectional (Spain)	91	50	80(87.9)	507	48 (52.7%)	81 (89%)	0	*n* = 16 (17.6%)	*n* = 66 (72.5%)	*n* = 52 (57.1%)	Average BMI 25.3	*n* = 63 (69.2%)	Low ABI (*n* = 4) in men > 45yrs of age with 3 or more CVD risk factors (*n* = 3), 1 defaulted ART but all exposed to protease inhibitors
2008 Palacios R et al.[27]	Descriptive cross-sectional (Spain)	99	58.7	88(82.8)	482	91 (91.9)	*n* = 99	99	31 (31.3)	30 (30,3)	36 (36.4%)	MS 39 (39.4%)	*n* = 68 (69.4)	HIV infection increases risk for PAD (RR of 1.91). PLWH with PAD were less likely to smoke tobacco (RR 0.69). Risk factors for PAD in PLWH were dyslipidaemia (RR 2.00) and diabetes mellitus (RR 1.63) All PLWH with low ABI had conventional cardiovascular risk factors
2008 Periard D et al.[28]	Cross-sectional descriptive observational study (Switzerland)	92	49.5	70 (76.1)	CD4 + T cell count 200–500 cells/ul in 35 (38%), > 500 cells/ul in 51 (55.4%);	73 (79.3)	92	32	4 (4.3)	57 (62)	25 (27.2)	25 (27.2)	12 (13)	High prevalence of PAD in PLWH (20.7%) with an early presentation in PLWH age (median age 49.5yrs). PAD associated with age (OR 1.09), diabetes mellitus (OR 13.5), smoking (OR 1.7), and low CD4 + T cell count (OR 27.2) in multivariate analysis
2009 van Marle J et al.[23]	Cross-sectional descriptive clinical audit (South Africa)	154	41.4	71(46.1)	CD4 + T cell count < 200 cells/uL 57(38%); 200–500 cells/uL 31 (47.5%); >500 cells/uL21 (13.5%)	NS	5 (3.2)	0	5 (5.5)	72 (79)	17 (18.7)	Obesity *n* = 0	2 (2.2)	PAD associated with tobacco use, diabetes and CD4 + T cell count < 200 cells/ul. Fewer traditional risk factors recorded in PLWH than in PWoH. Clinical presentation gangrene/ulcers *n* = 88 (57.1%); rest pain *n* = 51 (33%) High rate (31.9%) of primary amputation with low salvage rate (36.1%) and secondary amputations; Mortality (15.4 months mean) *n* = 31 (20%). Hypoalbuminaemia a predictor of poor surgical outcome.
2010 Johns K et al.[29]	Cross sectional, descriptive (Canada)	167	52	163 (97.9)	NS	NS	163 (97.9)	0	44 (26.3)	101 (60.6)	68 (40.7)		130 (77.8)	Prevalence of PAD low in PLWH. PAD occurred in presence of traditional risk factors
2010 Jang JJ et al.[30]	Cross sectional descriptive study (United States)	102	48.4	58(57)	565.4	mean viral load 10856.5 cps/ml	NS	0	12 (12)	76 (75)	29 (28)	26 (25)	23(23)	Elevated osteoprotegerin was associated high ABI. Low ABI in younger PLWH associated with traditional cardiovascular risk factors and with inflammation (increased C-reactive protein, interleukin 1β)
2010 Choi AI et al.[31]	Longitudinal in veterans cohort (multicenter, United States)	17,264	46.4	17,091(97)	367.5	mean viral load log 3.5cps/ml	13,889 (80.5)	0	1304 (7.55)	3738 (21.7)	6073 (35.2)	Median BMI 25	2857 (16.6)	PAD *n* = 118; Risk factors for PAD on multivariate analysis estimated glomerular filtration rate (eGFR) < 30 mL/min (HR 1.88); albuminuria (HR 3.45) or both (HR 4.05). Poor kidney function is a significant risk factor for PAD in PLWH
2010 Ye, Y et al.[32]	Cross-sectional descriptive study (China)	82	Mean 23.7	50 (61)	404.7	mean viral load log 2.66cps/ml	41 (50)	43	NS	41 (50)	NS	Mean BMI 23.7	NS	Factors associated with reduced ABI were age (B = 0.03) and HIV infection (B = 0.069) although HIV factors not significantly associated with reduced ABI
2011 Qaqa AY et al.[33]	Prospective longitudinal (United States)	113	47.1	68 (60)	< 200 *n* = 24 (21.1%)	67 (59.3)	113(100)	0	13 (11.5)	73 (64.6)	38 (33.5)	25 (22.1)	20 (17.7)	PAD in PLWH only significantly associated with male sex (RR 4.15); no association between PAD and conventional cardiovascular risk factors in this cohort
2011 Choi AI et al.[34]	Longitudinal, descriptive cohort (multicentre, United States)	10,931	46.7	10,647 (97.4)	283.77	120.6	100% naïve at study entry	0	1869 (17.1)	5466 (50)	4471 (40.9)	median BMI 25	587 (53.7)	PAD developed in 79 PLWH (0.72%). Neither tenofovir or abacavir therapy was linked to the risk of development of PAD (HRs 0.70 and 1.00) respectively in adjusted model).
2013 Esser S et al.[35]	Longitudinal, descriptive cohort study (HIV Germany)	803	44.2	670 (83.4)	461	median viral load 4.2 Log cps/ml	683 (85)	0	41 (5.1)	571 (71)	169 (21)	Obesity 58 (7.2)	516 (64.3)	PAD developed in 23 PLWH (2.9%). Risk factors for cardiovascular disease were reported with both age and time since HIV diagnosis (OR 2.2 and 1.2 per decade respectively), symptomatic HIV infection (OR 2.6), smoking (OR 5.96) and diabetes mellitus (OR 5.96) independently associated with CVD in multivariate analysis.
2014 Schouten J et al.[36]	Longitudinal, prospective descriptive study (Netherlands)	540	52.9 (median)	476 (88)	565	516 (98.5)	540 (100)	524	NS	*n* = 362 (67%)	*n* = 245 (45.4%)	Obesity *n* = 43 (8%)	NS	Significantly higher mean number AANCCs in PLWH than controls, PAD higher in PLWH (2.6% PLWH vs. 0.6% PWoH); AANCCs associated with age (OR 1.39), smoking (OR 1.08), positive family history of CVD (OR 1.88), known cumulative duration of immunodeficiency per year with CD4 + T cell count < 200 cells/µl (OR 1.23).
2014 Kwiatkowska W et al.[37]	Descriptive cross-sectional study (Poland)	111	42	71 (64)	546	aviraemic 28 (25)	111 (100)	40	3 (2.7)	86 (77.5)	47 (42.3)	metabolic syndrome 22 (19.8)	77 (69.4)	Overall prevalence of PAD in PLWH was low although ABI was not normal in ~ 20%. Abnormal ABI not linked to viral factors or ART regiment
2015 Knudsen A et al.[38]	Descriptive cross-sectional study (Denmark)	102	52	77(75)	644	viral load median 19cps/ml	96 (95)	0	7 (7)	58 (58)	28 (28)	mean body-mass index 26	NS	Low prevalence of PAD in PLWH. Abnormal post-exercise ABI correlated significantly with BMI and tended to correlate with Framingham risk score. Viral factors had no obvious influence on post-exercise ABI
2018 Hinojosa CA et al.[39]	Descriptive longitudinal cohort (Mexico)	206	44	168 (81.6)	481	mean viral load 11178,12 cps/ml	206 (100)	0	22 (10.7)	55 (26.7)	24 (11.65)	NS	NS	Abnormal ABI in 20%. Abnormal ABI not statistically significantly related to traditional CVD risk factors. Significant factors associated with abnormal ABIs - viral load and the number of years with HIV/AIDS (both *p* = 0.04).
2018 Kamdem F et al.[40]	Descriptive cross-sectional study (Cameroon)	144	46	40 (28)	451	aviraemic 66 (46%)	128 (88.9)	0	5 (3.5)	6 (4.2)	29 (20)	36 (25)	44 (30.6)	Lower than expected prevalence of PAD (6.9%). Low ABI significantly associated with advanced HIV disease (WHO stage III/IV) adjusted OR of 11.1. ART appeared to be protective (OR 0.18)
2018 Knudsen AD et al.[41]	Longitudinal descriptive cohort (Denmark)	908	median 52	770 (85)	670	aviraemic 860 (95)	889 (98)	11,106	47 (5)	597 (65.7)	415 (48)	Obesity 95 (11)	219 (26)	PAD diagnosed in 112 participants − 7 (0.8%) with symptomatic PAD. Higher prevalence of PAD in PLWH. In univariate analysis, PAD risk increased in HIV infection (OR 2.4), with increased age (OR 1.4); diabetes mellitus (OR 2.0); current smoking (OR 4.3); hypertension (OR 2.1); Kidney dysfunction (OR 1.1). Female sex associated with PAD in adjusted analysis. Viral factors (viral load, CD4 + T cell count or presence of AIDS) showed no significant association with PAD
2018 Beckman JA et al.[42]	Retrospective longitudinal cohort (multicenter, United States)	28,714	median 48	27,853 (97)	median 389	median viral load 1320cps/ml	11 974 (41.7)	63,239	2441 (8.5)	20186 (70.3	14,357 (50)	4451 (15.5)	2182 (7.6)	Risk factors for PAD include HIV infection (HR 1.2), age (HR 1.57), uncontrolled hypertension (HR 1.53), diabetes mellitus (HR 1.92), current smoking (HR 1.61), elevated triglycerides (HR 1.13), low CD4 + T cell count (HR 1.1) and HIV viraemia (HR 1.05). CD4 + T cell count < 500 cells/µl associated with a 50% 4-year mortality after incident PAD.
2019 Rosenson RS et al.[43]	Descriptive audit of the marketscan database (United States)	82,426	19–44	69,137 (83.9)	NS	NS	29,129 (96)	329,704	6664 (8.1)	5640 (6.8)	23,740 (28.8)	NS	Statin use 15,619 (18.99)	Rate for PAD in PWH was 0.65 per 1000 person years (vs. rate of 0.31 in PWoH). Risk factor for PAD was HIV infection (HR 1.96). The risk of hospitalisation with lower extremity atherosclerotic disease in PLWH was higher in patients with diabetes mellitus and liver disease
2019 Agu CE et al.[44]	Descriptive cross-sectional study (Nigeria)	150	42.7	41(27.2)	505	mean viral load 40.95 cps/ml	100	50	6 (4%)	NS	24 (15.9)	26 (15.3)	NS	Higher rates of PAD (14.6% vs. 2%) and MS in PLWH compared with PWoH. Factors significantly associated with risk of PAD in PLWH were increased total cholesterol (OR 1.78) and increased LDL (OR 1.82). Significant negative correlation between PAD and duration of ART (*r*= −0.163, *p* = 0.04)
2019 Cedarbaum E et al.[45]	Cross sectional cohort (Women’s Interagency HIV Study - WIHS, multicenter, United States)	1381 (HCV *n* = 290)	median 49.5	1381 (100)	median 590	939 (68)	1221 (88.4)	424	295 (21)	544 (39.3)	736 (53.2)	Median BMI 29.4	Statin use *n* = 201 (14.6%)	Factors associated with PAD in multivariate analysis were older age (OR 2.01), smoking (OR 1.27) and higher systolic blood pressure (OR 1.14); black race (OR 2.3) HIV and HCV infection are not significantly associated with greater PAD risk relative to uninfected women with similar risk factors
2019 Aurpibul L et al.[46]	Descriptive, cross-sectional cohort study (Thailand)	362	57.8	155(43)	491	VL > 400 cps/ml (1)	362	362	39 (11)	54 (15)	143 (40)	105 (29)	149 (41)	Risk factors for PAD in PLWH were female sex (OR 2.09) and low BMI (OR 1.73). Prevalence of PAD in older PLWH was relatively low. Low ABI detected more commonly in PWoH (*n* = 25 − 7%)
2019 Aurpibul L et al.[47]	Descriptive, cross-sectional cohort study (Thailand)	107	59.7	47(45)	456	107 (100)	107 (100)	48	12 (11)	14 (13)	48 (45)	Obese 14 (13)	46 (43)	No difference in ABI or CAVI in PLWH vs. PWoH. Abnormal CAVI significantly associated with diabetes mellitus only (OR 1.54). Low ABI in 4 PWoH
2019 Alonso A et al.[48]	Retrospective record audit (United States)	19,798	43	16,036 (81)	NS	NS	NS	59,302	1951 (10)	1940 (10)	5198 (26)	1130 (6%)	5432 (27)	PAD *n* = 394 (2%) in PLWH (incidence rate of 0.9). HR of 1.4 in model adjusted for age and sex for PAD in PLWH.
2019 Masiá M et al.[49]	Descriptive longitudinal cohort (Spain)	9712	median 36	7986 (82)	368	Median VL 48 218 cps/ml	0 (0)	0	NS	2379 (24.5)	853 (9)	NS	NS	Incidence of PAD within the CoRIS cohort was too low for inference of risk
2020 Kim TI et al.[50]	Retrospective database review (United States)	5154	55.74	NS	NS	NS	NS	1,667,466	1566 (30.39)	*n* = 1818 (35.27)	1013 (19.65)	NS	2030 (39.38)	Significant increase in PLWH with PAD undergoing lower limb revascularisation between 2003 and 2014 (0.21% to 0.52%, *p* < 0.01). PLWH with PAD more likely to be younger, male, and have fewer comorbidities, at the time of intervention compared to PWoH. PLWH more likely to have open surgery but HIV not associated with significantly increased need for amputation.
2020 Rerkasem A et al.[51]	Descriptive, cross-sectional cohort study (Thailand)	789	43.2	399 (50.6)	546	700 (89.9%)	789 (100)	41	48 (6.1)	239 (30.3)	166 (21)	mean BMI 22.4	180 (22.8)	Reduced ABI in both groups at 1 year but steeper decline in ABI in PLWH; Higher rate of subclinical atherosclerosis in PLWH than PWoH with end-stage renal failure
2021 Desormais I et al.[52]	Descriptive cross-sectional cohort (Burundi)	287	50.9	54 (18.8)	431.7	263 (91.6)	287 (100)	0	17 (5.9)	6 (2.1)	43 (14.9)	Obese 38 (13)	Elevated cholesterol 14 (4.9)	Factors associated with PAD on multivariate analysis in PLWH were hypertension (OR 2.42), and stage IV HIV clinical infection (OR 4.92); Association with viral load and smoking not statistically significant
2022 Tran ML et al.[53]	Retrospective record review (National In-patient Sample (NIS) database 2003-17, United States)	1264	63.8	818 (64.4)	NS	NS	NS	223,648	364 (28.8)	570 (45.1)	594 (47)	NS	334 (26.4)	Higher rate of critical limb ischaemia in PLWH who were symptomatic compared to asymptomatic PLWH or PWoH (66.2% vs. 46.3% and 43.6%; *P* < 0.01). PLWH more likely to require open revascularisation or amputations than PWoH. In-hospital mortality rate was higher in PLWH who were symptomatic. PAD in PLWH occurred at younger age with fewer risk traditional factors.
2022 Suarez-Zdunek MA et al.[54]	Prospective longitudinal cohort (COCOMO cohort - Copenhagen comorbidity in HIV infection; Denmark)	844; HCV co-infection 47 (5.6)	50	725 (86)	667	801 (95)	832 (98.6)	0	33 (3.9)	520 (61.6)	339 (40.2)	Obesity 88 (10.4)	383 (45.4)	Risk factors for PAD in PLWH in multivariate analysis diabetes mellitus (RR 4.33); CD4 + T cell count < 350cells/µl (RR 2.66); each decade of ART doubled risk of PAD (RR 1.88 -significant in univariate analysis only). Viral load was not significantly associated with de novo PAD. Biomarkers associated with PAD hs-CRP (RR 1.33 for each doubling) and IL 6 (RR 1.32 for each doubling). CD14, CD163 and current HCV co-infection was not associated with PAD.
2022 Teng AE et al.[55]	Retrospective review of database (United States)	1945	55.5	1405 (72.2)	NS	NS	NS	345,879	683 (35.2)	914 (47%)	NS	Obesity 150 (7.7)	NS	HIV status did not impact amputation; in-hospital mortality was similar between groups. PLWH were younger and had fewer traditional risk factors
2023 Bundgard J et al.[56]	Descriptive, longitudinal cohort (Denmark)	1012	50	863 (85)	687	950 (94)	1012 (100)	57	42 (4)	643 (63.5)	406 (40)	Median BMI 24.5	458 (45)	PLWH: Higher concentrations of soluble thrombomodulin (*P* = 0.03) and syndecan-1 (*P* < 0.001) and lower concentration of sCD40L (*P* < 0.001) compared with controls. High concentration of soluble thrombomodulin, but not syndecan-1 or sCD40L, was associated with lower odds of PAD (OR 0.58) in PLWH at baseline only.
2023 Kentoffio K et al.[57]	Retrospective audit of database (United States)	357	72.1	261 (73.1)	NS	NS	NS	168,196	200 (56)	144 (40.34)	317 (88.8)	Obesity 57 (15.97)	271 (75.91)	HIV was associated with two-fold increased risk of MALE (amputation or thrombosis and embolism) (HR 1.26 in multivariate analysis) amputation or thrombosis and embolism; Cumulative incidence over study period was 24.6% in PLWH and 14.5% in PWoH
2023 Yeboah K et al.[58]	Descriptive case-control study (Ghana)	308	38.6	102 (33.1)	Median; 416.5	NS	158 (51.3)	156	Diabetics excluded	62 (20.1)	114 (37)	Obesity 42 (13,6)	NS	ART-naïve PLWH had a higher risk of PAD compared to PWoH and ART-treated PLWH (OR 1.9). CD4 + T cell count < 200 cells/µl was significantly associated with increased risk of PAD (OR 3.68) and in cART treated PLWH, tenofovir and efavirenz were both associated with increased risk of PAD (OR of 5.76 and 9.28)
2023 Rerkasem et al.[59]	Descriptive, cross-sectional cohort study (Thailand)	892	42.87	458 (51)	525.3 (median)	789 (88.5)	892	0	51 (5.7)	270 (30.3)	191 (21.4)	Mean BMI 22.3	207 (23.2)	PAD prevalence of 4.37%. Independent risk factors for PAD: female sex (OR 2.86), < 30 years of age (OR 4.66), Obesity (OR 3.53), being underweight (OR 1.55) and having detectable HIV-1 RNA ≥ 20 copies/mL (OR 1.85). PAD appears to be relatively poorly correlated with traditional risk factors of CVD.

*µL* microliter, *AANCC* age−associated non−communicable comorbidities, *ABI* ankle−brachial index, *AIDS* acquired immunodeficiency syndrome, *ART* antiretroviral treatment, *BMI* body−mass index, *cps/ml* copies per millilitre, *CVD* cardiovascular disease, *eGFR* estimated glomerular filtration rate, *HCV* Hepatitis C virus, *HIV* human immunodeficiency virus, *HR* hazard ratio, *HT* hypertension, *LDL* low density lipoprotein, *MALE* major adverse limb event, *MS* metabolic syndrome, *n* number, *NS* not stated, *OR* odds ratio, *PAD* peripheral arterial disease, *PLWH* people living with HIV, *PWoH* people without HIV, *RNA* ribonucleic acid, *RR* Relative Risk, *VL* viral load, *WHO* World Health Organisation, *yrs* years

**Fig. 2:**
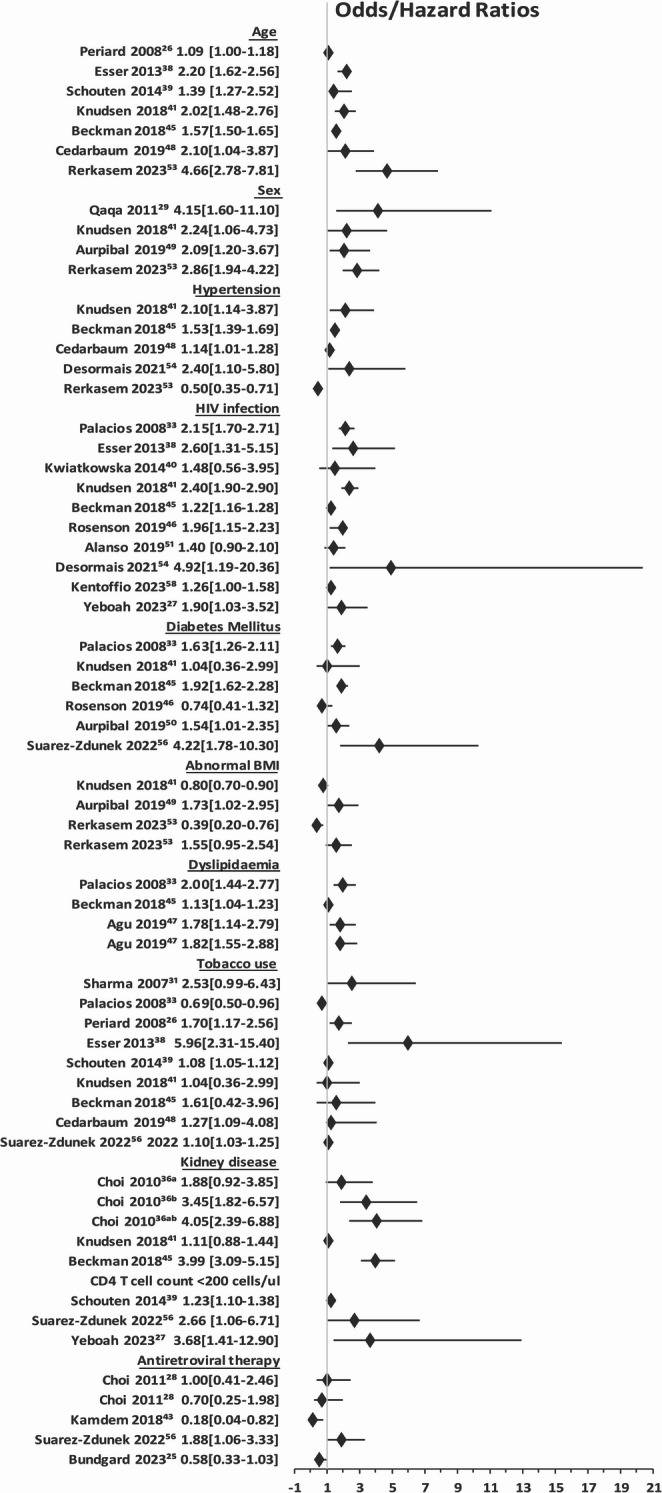
Summary figure of significant risk factors associated with development of peripheral arterial disease in people living with HIV

A summary of the risk factors which were statistically significantly (*p* < 0.05) associated with PAD in PLWH is included in the forest plot in Fig. 2 . For comparative purposes, relative risks were converted to odds ratios with an estimated baseline prevalence rate of 10% and hazard ratios were reported directly. The most common risk factors for PAD in PLWH were the presence of HIV, tobacco use and increased age. Only certain studies reported the presence of traditional cardiovascular risk factors (tobacco smoking, hyperlipidaemia/dyslipidaemia, diabetes mellitus, hypertension and a family history of cardiovascular disease) and in these studies, there was heterogeneity in the description of these factors. Dyslipidaemia is a recognised effect of protease inhibitors but abnormal lipid profiles were reported variously as dyslipidaemia [60], high total cholesterol [44] and high low density lipoprotein [44] and in some cases, patients were reported to have a metabolic syndrome [27, 37]. Tobacco use was identified as a risk factor in 9 studies with an OR ranging from 1.08 [36]− 5.96 [35] and one paper stating that PLWH, included in the study, were less likely to smoke than PWoH (OR – 0.67) [27]. Hypertension and diabetes mellitus were less frequently described although in some studies, the presence of diabetes mellitus was an exclusion factor [61]. Similarly, HIV-related risk factors for PAD (previous World Health Organisation stage III or IV disease, low CD4 + T cell count, a previous diagnosis of AIDS and presence of detectable viraemia) were reported inconsistently in the cohort with 31 studies (81,5%) providing viral load, treatment status and CD4 + T cell counts for PLWH. The differences in the sample sizes may account for the differences in the significance of risk factors as well as the wide confidence intervals reported. Importantly, several studies were reviews of large databases including the veterans cohort [31, 42], the Marketscan Insurance Database [43, 48], the Medicare fee For Service Database - Centres for Medicade and Medicare services [57] and the National In-patient Database [50, 53, 55]. In these cases, certain data were missing including HIV-related risk factors like CD4 + T cell count and viral load. For this reason, it was not possible to weight risk factors by numbers of participants (Fig. 2).

The outcomes of intervention (open surgical and endovascular revascularisation and requirements for amputation) were reported in 7 (18,4%) of studies. Hepatitis C co-infection was reported in 2 studies [45]. Hypoalbuminaemia and renal dysfunction were associated with PAD development or outcomes in 3 studies [23, 34, 41]. No other comorbid infections were described in these studies. Only 7 studies were conducted in sub-Saharan Africa [22–24, 40, 44, 52, 61].

## Discussion

Peripheral arterial disease remains relatively understudied in PLWH although the morbidity and mortality are high particularly in patients with CLTI and those requiring amputation [4]. In sub-Saharan Africa, there are high prevalence rates of diabetes mellitus, hypertension and obesity and increasing numbers of people smoking tobacco together with an aging population which includes PLWH [44, 62–64]. Outcomes of interventions for PAD are typically inferior in PLWH with higher rates of amputation following revascularisation and higher mortality particularly in PLWH who have poor virologic control [3, 50, 57, 65].

The majority (24) of studies reviewed indicated a relationship between HIV infection and the development of PAD but there was limited information on the numbers of individuals with CLTI and these are the patients which would require intervention for the disease. Study design included retrospective database reviews [3, 43, 50], longitudinal cohorts [25], case-control studies [27, 28, 39] and small clinical cohorts [22–24] which makes generalisation of the findings difficult and may explain the heterogeneity in the risk factors identified. Participant inclusion criteria varied with at least two studies enrolling only women as part of the Women’s Interagency HIV Study [25, 45]. Increasing age, HIV infection and tobacco smoking were the most commonly identified factors associated with PAD across the studies identified.

Few studies have assessed the outcomes in PLWH with PAD in sub-Saharan Africa following wide-scale ART implementation. The impact of ART on PAD risk appears contradictory and studies reported that drugs including efavirenz and tenofovir did not increase the risk [34], were protective [40] or substantially increased the risk [58] of PAD. Dolutegravir, an integrase inhibitor, is first-line ART in many countries in Africa and is associated with increased weight gain. Some studies have considered the impact of dolutegravir on cardiometabolic risk factors and early results are encouraging [66, 67] but it remains to be seen whether there will be any effect on the prevalence of PAD in an African population.

Most of the studies were descriptive and observational and the importance of management of risk factors and the identification of biomarkers was outside their scope. Studies looking at coronary and cerebrovascular atherosclerosis have emphasised the importance of early patient identification and secondary prevention strategies to reduce morbidity and mortality [68]. The American Heart Association guidelines have published statements on prevention of cardiovascular disease in PLWH and recommend active management both of traditional risk factors and managing HIV-related risk factors (including early initiation of ART) [68]. Although there is likely to be overlap between the pathogenic factors driving coronary and cerebrovascular atherosclerosis and PAD, further mechanistic studies are needed. Biomarkers of immunothrombosis and thromboinflammation including proinflammatory cytokines, markers of endothelial activation and dysfunction and coagulation factor dysregulation were inconsistently assessed in the reported studies although both processes are pathogenic in arterial disease in PLWH [11] and may be used to guide management decisions [14]. Lipoprotein-associated phospholipase A_2_, for example has been included as a non-traditional cardiovascular risk factor by the American Association of Clinical Endocrinologists/American College of Endocrinology guidelines for lipid management particularly in PLWH [69]. Inflammatory biomarkers like hs-CRP, soluble thrombomodulin, syndecan and soluble CD40L were included in two studies only and remain to be evaluated in larger cohorts of PLWH with PAD [54, 56].

Coronary atherosclerosis, although more common in Black Africans living with HIV and increasing in prevalence [70, 71], is generally less common in Africans suggesting that there are unique factors which predispose this population to the development of PAD. Although the prevalence of ischaemic heart disease has increased by over 70% in the last 3 decades, the prevalence of PAD has increased by over 150% in sub-Saharan Africans over the same period [72].

Importantly, there are relatively few studies in sub-Saharan African cohorts with PAD and the contribution of PAD to HIV-related morbidity and mortality remains unclear. Studies of the pathogenesis of this important non-communicable disease complication in Black Africans living with HIV is likely to reveal both novel biomarkers to predict disease development and novel therapeutic targets.

## Conclusions

PAD is a non-communicable complication in PLWH but has been relatively understudied in this population. Additional studies are required to assess pathogenic factors underlying the development of this disease, to identify additional risk factors for PAD in aging PLWH and to develop new diagnostic and prognostic biomarkers which may lead to new therapies to reduce morbidity and mortality.

## Key References

- Beckman JA, Duncan MS, Alcorn CW, So-Armah K, Butt AA, Goetz MB, et al. Association of Human Immunodeficiency Virus Infection and Risk of Peripheral Artery Disease. Circulation. 2018;138(3):255 − 65.10.1161/CIRCULATIONAHA.117.032647
  - ○ A large multicenter study looking at the association of HIV with peripheral arterial disease after controlling for traditional cardiovascular risk factors.
- (Bundgard J, Jensen AMR, Suarez-Zdunek MA, Hogh J, Gerstoft J, Benfield T, et al. Peripheral Artery Disease and Markers of Endothelial Dysfunction and Platelet Activation in People With HIV. J Acquir Immune Defic Syndr. 2023;93(3):237 − 43.10.1097/QAI.0000000000003194
  - ○ Recent laboratory analysis of biomarkers of endothelial dysfunction in people living with HIV and PAD.
- Johnston LE, Stewart BT, Yangni-Angate H, Veller M, Upchurch GR, Jr., Gyedu A, et al. Peripheral Arterial Disease in Sub-Saharan Africa: A Review. JAMA Surg. 2016;151(6):564 − 72.10.1001/jamasurg.2016.0446
  - ○ Major review of arterial disease in sub-Saharan Africa.
- Kentoffio K, Sun T, Xu J, Parikh RV, Hsue PY, Secemsky EA. Longitudinal outcomes following peripheral vascular intervention among older persons living with HIV. Vasc Med. 2023;28(6):564 − 70.10.1177/1358863X231191822
  - ○ A large review of the MEDICARE database and reviewing both risk factors and intervention outcomes in people with and without HIV and with peripheral arterial disease.
- Mayne ES, Louw S. Good Fences Make Good Neighbors: Human Immunodeficiency Virus and Vascular Disease. Open Forum Infect Dis. 2019;6(11):ofz303.10.1093/ofid/ofz303
  - ○ Comprehensive review of markers of endothelial dysfunction in people living with HIV.
- Ombajo LA, Penner J, Nkuranga J, Omodi V, Otieno E, Ndinya F, et al. Change in Blood Pressure, Weight, and Other Cardiovascular Disease Risk Factors After Switch From Protease Inhibitors to Dolutegravir: Post hoc Analysis of the 48-week Randomised Second-line Switch to Dolutegravir Trial. Clin Infect Dis. 2025;80(4):889 − 92.10.1093/cid/ciae514
  - ○ Important study examining the effect of the change in antiretroviral therapy regimens to include dolutegravir and the impact on cardiometabolic health.
- Shehu MN, Adamu UG, Ojji DB, Ogah OS, Sani MU. The Pandemic of Coronary Artery Disease in the Sub-Saharan Africa: What Clinicians Need to Know. Current Atherosclerosis Reports. 2023;25(9):571 − 8.10.1007/s11883-023-01136-9
  - ○ A review on the changing epidemiology of both traditional and non-traditional cardiovascular risk factors in Africa.
- Yeboah K, Musah L, Essel S, Agyekum JA, Bedu-Addo K. Asymptomatic peripheral arterial disease in HIV patients in Ghana: A case-control study. J Vasc Nurs. 2023;41(4):203 − 8.10.1016/j.jvn.2023.07.001
  - ○ Clinical study of asymptomatic PAD in sub-Saharan people living with HIV.

## Data Availability

No datasets were generated or analysed during the current study.
